# Impact of genetic risk score on the association between male childlessness and cardiovascular disease and mortality

**DOI:** 10.1038/s41598-021-97733-2

**Published:** 2021-09-17

**Authors:** Angel Elenkov, Olle Melander, Peter M. Nilsson, He Zhang, Aleksander Giwercman

**Affiliations:** 1 Reproductive Medicine Centre, Skåne University Hospital, Malmö, Sweden; 2 Department of Translational Medicine, Clinical Research Centre, Lund University, Jan Waldenströms gata 35, Building 60, Plan 9, 20502 Malmö, Sweden; 3 Department of Clinical Sciences Malmö, Lund University, Malmö, Sweden; 4 Department of Emergency and Internal Medicine, Skåne University Hospital, Malmö, Sweden

**Keywords:** Cardiology, Cardiovascular genetics, Testis, Risk factors

## Abstract

Childless men are reported to have a higher risk of cardiovascular disease (CVD) and mortality. Information on inherited genetic risk for CVD has improved the predictive models. Presuming that childlessness is a proxy of infertility we aimed to investigate if childless men inherit more often genetic traits for CVD and if combining genetic and parenthood information improves predictive models for CVD morbidity and mortality. Data was sourced from a large prospective population-based cohort where genetic risk score (GRS) was calculated using two sets of either 27 (GRS 27) or 50 (GRS 50) single nucleotide polymorphisms (SNPs) previously found to be associated with CVD. Part of the participants (n = 2572 men) were randomly assigned to a sub-cohort with focus on CVD which served as an exploratory cohort. The obtained statistically significant results were tested in the remaining (confirmatory) part of the cohort (n = 9548 men). GRS distribution did not differ between childless men and fathers (p-values for interaction between 0.29 and 0.76). However, when using fathers with low GRS as reference high GRS was a strong predictor for CVD mortality, the HR (95% CI) increasing from 1.92 (1.10–3.36) for GRS 50 and 1.54 (0.87–2.75) for GRS 27 in fathers to 3.12 (1.39–7.04) for GRS50 and 3.73 (1.75–7.99) for GRS27 in childless men. The confirmatory analysis showed similar trend. Algorithms including paternal information and GRS were more predictive for CVD mortality at 5 and 10 years follow-ups when compared to algorithms including GRS only (AUC 0.88 (95% CI 0.84–0.92) and 0.86 (95% CI 0.84–0.90), and, AUC 0.81 (95% CI 0.75–0.87) and 0.78 (95% CI 0.73–0.82), respectively). Combining information on parental status and GRS for CVD may improve the predictive power of risk algorithms in middle-aged men. Childless men and those with severe infertility problem may be an important target group for prevention of CVD.

## Introduction

Approximately 10–15% of all couples in reproductive age are involuntarily childless^1^. However, as biological parenthood is dependent on the fertility status of both partners, ‘male infertility’ is not a well-defined clinical entity, but rather a categorisation based on laboratory, clinical or epidemiological criteria. Poor semen quality is a laboratory marker of impaired male fertility, whereas in the clinical setting, lack of signs of female reproductive dysfunction is regarded as an indicator of male contribution to a couple’s infertility. In an epidemiological context, male childlessness is often used as proxy for male infertility^2–5^.

Available data point to a higher risk of developing various non-communicable adult-onset diseases among infertile men. Low sperm counts in the ejaculate have been associated with higher risk of metabolic syndrome (MetS), type 2 diabetes and all-cause mortality^6–9^. In a population-based register study, more frequent prescription of drugs used for the treatment of hypertension, dyslipidemia, and MetS was found in men who fathered children following intracytoplasmic sperm injection (ICSI), the preferred method of symptomatic treatment of infertility associated with poor semen quality^10^, as compared to those who became fathers without assisted reproduction. Married, childless men, were reported to have a higher risk of cardiovascular disease (CVD), type 2 diabetes^3,4^ and death caused by CVD^2^. Therefore, a man’s reproductive potential has been suggested to be a marker of general health^9^.

CVD is the leading cause of morbidity and mortality in the US as well as in the European Union to a cost of hundreds of billion €, and responsible for 400,000 annual US deaths and 1.8 million EU deaths^11–13^. Therefore, improving CVD risk prediction and prevention is an important public health goal and is embedded in the Action Plan for the Prevention and Control of Non-Communicable Diseases in the WHO European Region for the time period 2016 to 2025^14^.

Current risk assessment algorithms for CVD most often incorporate information about gender, age, BMI, blood pressure and cholesterol levels, as well as smoking and diabetes. The clustering of CVD in families has been well documented since the early 1930s, and in recent years, in order to improve the predictive effect of the available models, a genetic risk score (GRS) for CVD has been developed^15^. This GRS is based on the aggregated effects of multiple single nucleotide polymorphisms (SNP) identified by genome-wide association studies^15^, each of which has a small effect size. A large population-based study from Sweden have shown high predictive value of GRSs in relation to incident fatal and non-fatal CVD, independently of the traditional risk factors including self-reported family history of myocardial infarction. Thus, applying these GRSs can improve the reliability of risk estimates for cardiovascular morbidity and mortality, especially for young people^13,15^.

The clinical and epidemiological link between a man’s reproductive function and his general health has been suggested to be influenced by an interplay between lifestyle, environmental and genetic factors, some of them acting already at the stage of foetal development^16–19^.

Therefore, our *first aim* was to test if aggregated genetic risk factors for coronary artery disease (CAD), i.e. the most common clinical entity of CVD, also constitute a part of the aetiology of male infertility and, if not, to assess whether there is an interaction between the two factors—genetic predisposition and infertility—in prediction of incident coronary events and CVD mortality. *Secondly,* we aimed to answer a clinically relevant question—to which extent can CVD risk assessment be improved by combining information on paternal status and GRS. For this purpose, we used data from a population-based study, specifically designed to study the epidemiology of CVD, among middle-aged men from Sweden using information on paternal status in late mid-life as a fertility proxy. Childless men have already been shown to be at higher risk for CVD and associated mortality in a similar cohort from the same population^2^.

## Subjects and methods

### Study population

#### Exploratory cohort

The Malmö Diet and Cancer (MDC) study is a population-based cohort of 30,446 Malmö residents (12,120 men born between 1926 and 1945, and 18,326 women born between 1923 and 1950) enrolled during 1991–1996^20^. After random selection among MDC participants 2572 men were subsequently included in a sub-cohort named MDC—Cardiovascular Cohort (MDC-CVC) which was aimed to study prevalence and incidence of CVD. MDC-CVC was used as exploratory cohort in the present study.

At baseline all MDC-CVC participants completed a questionnaire regarding marital status, number of children, and lifestyle factors (alcohol (g/day), and smoking habits). The participants also underwent a clinical examination including weight, height, body composition, blood pressure measurement, collection of venous blood samples in a biobank (used subsequently for genetic analyses), blood lipids and blood glucose. A detailed description of the cohort has previously been presented^20^.

#### Confirmatory cohort

The male participants from the remaining part of the MDC (n = 9548) comprised a confirmatory cohort in the present study. From those men no information on levels of blood lipids and glucose were available.

The study has been approved by the Swedish Ethical Review Authority (LU 91-50) and complies with the declaration of Helsinki. Participants gave written informed consent.

### Information on childlessness

Gathering the information regarding childlessness was described previously^3^. In short, it came from two sources: the *baseline self-administered questionnaire* and the *Swedish Tax Agency (STA)*. The participants were stratified into four groups: ‘Childless’, ‘Fathers, ‘Conflicting information’, or ‘Unknown’. ‘Conflicting information’ appeared if a participant answered ‘No’ to ‘*Do you have any children*?’ in the baseline questionnaire, but was registered with one or more children in the STA. Men who answered “yes” in the questionnaire and/or had children registered in the STA were categorized as “Fathers”. Men with ‘Conflicting information’, who—according to STA—became fathers after the entry date into the MDC-CVC cohort, were treated as ‘Childless’ as they were childless at baseline. The men with ‘Conflicting information’ who were registered as fathers in the registry of the STA before the entry date were treated as ‘Fathers’. ‘Unknown’ was used in cases when no information regarding children was available from the STA and if no answer was provided to ‘*Do you have any children*?’ in the baseline questionnaire.

### End points

The primary endpoints in the prospective analysis were time to occurrence of:

1. Coronary artery disease (CAD), and
2. Death due to CVD.

Information on the end points during follow-up was obtained through cross-linking the cohort members with the following population registers: The Swedish National Hospital Discharge Register, the Swedish National Coronary Angiography and Angioplasty Registry (SCAAR), the Swedish National Cause of Death Register (SNCDR) and the National Swedish Classification Systems of Surgical Procedures. The registers have recently been validated for classification of outcomes and found to be of high quality with validity 86–97%^21^. Information on CVD mortality including age and date of death were retrieved from SNCDR and was defined as any of ICD-9 codes 390–459 or ICD-10 codes I00-I99 as the main cause of death in the SNCDR.

Incident CAD was defined as fatal or non-fatal myocardial infarction (ICD-9 code 410 and ICD-10 code I21), death due to coronary heart disease (codes 412 or 414 in ICD-9 or I22–I23 or I25 codes in ICD-10 in the SNCDR), or coronary artery bypass graft surgery (CABG) identified from national Swedish classification systems of surgical procedures and defined as procedure codes 3065, 3066, 3068, 3080, 3092, 3105, 3127, or 3158 (the Op6 system) or procedure code FN (the KKÅ97 system) or percutaneous coronary intervention (PCI) identified the SCAAR^22^. End points were registered until December 31st 2018.

### Modeling of genetic risk score

The association between CAD and mortality was investigated for two sets of GRSs. One GRS is the 27-SNP GRS (GRS27) described by Mega et al.^23^, and a second GRS based on the GRS27 SNPs plus 23 additional SNPs, in total 50 SNPs (GRS50) as previously done (Refs.^13,15^, Supplementary document) Each of the SNPs were previously shown to be associated with CAD. The GRS of each individual in the study was calculated as follows: the previously reported risk estimate for the modelled allele of each SNP was natural log-transformed and multiplied by two for homozygotes and with one for heterozygotes. The products were then summed. The mean (3.84 for GRS50) and standard deviation (0.43 for GRS50) of the study population were used to standardize each GRS to have a mean of 0 and one SD as unit of variance. Genetic risk was analyzed per standard deviation increment of the standardized GRS as well as by comparing those with *high* GRS (Quintile 5), with those with *intermediate* risk score (Quintiles 2–4), and those with *low* GRS (Quintile 1). The analysis for GRS 27 is provided as Supplementary document.

### Statistical analysis

Baseline characteristics for the study groups were presented using descriptive statistics as means (SD) and proportions. We used exploratory cohort (MDC-CVC) to test if aggregated GRS for CAD also predicts risk of male childlessness and to which extent can CVD risk assessment might be improved by combining information on paternal status and GRS.

The statistically significant results were tested in the confirmatory cohort.

In order to test our hypothesis that intermediate and high GRSs might be over-represented among childless men, logistic regression was performed for calculation of odds ratios (ORs) for each of these two scores using childless men vs. fathers as exposure and GRS category as outcome. Additional analysis was performed by linear regression using GRS as exposure, but as a continuous variable. In order to explore possible synergy in prediction of CVD related events, analysis for interaction between fatherhood (+/−) and GRS (three groups − low/intermediate/high) was included to the below-mentioned prospective analysis. Results are presented with p-values and bar plots.

Both for GRS27 and GRS50 the cohorts were divided into six different categories combining the fatherhood status (+/−) and the three groups of genetic risk (high–intermediate–low).

The category including “fathers with low GRS” was used as reference group.

CVD mortality risks were assessed using competing risk analysis as described by Fine and Gray^24^ incorporating death from non-CVD as competing risk. Results are shown as hazard ratios (HR) with age as underlying time scale.

In order to estimate the risks of incident CAD, an age-adjusted Cox regression was used. Kaplan–Meier plots allowed for visual evaluation of proportional hazards assumption.

In all statistical analysis mentioned above, the models were adjusted in two different ways due to some difference in the available variables for the two cohorts:

1. In the exploratory cohort the analysis was adjusted for marital status (married/unmarried/divorced/widower), educational attainment (no education/primary school/secondary school/high school/> 1-year education after high school/university degree), BMI in kg/m^2^, alcohol consumption in grams per day, smoking habits (regular smoker/occasional smoker/stopped smoking/never smoked), cholesterol and triglyceride levels (as continuous variable) and, finally, for prevalent diabetes (yes/no). Family history of myocardial infarction was introduced in the model as a score from 0 to 3 depending on the answer in baseline questionnaires. The score system was as follows: 0: No family history or no answer in baseline questionnaire; 1–3: a positive family history (myocardial infarction before the age of 60) from Father, Mother and Brother/Sister, respectively, contributing with one point each.
2. In the confirmatory analysis the model was adjusted for BMI, marriage status, smoking, educational level, prevalent diabetes (based on “yes” on the question “do you have diabetes” or intake of antidiabetic drug at baseline), family history of myocardial infarction but not alcohol consumption, triglyceride and cholesterol levels.

Additionally, in the confirmative cohort the area under the curve (AUC) values were analyzed in order to test the predictive effect of paternal information on the mortality estimates. In order to do this we analyzed two models:

- *Model 1* AUCs for a model adjusting for GRS 50 (continuous variable), age, BMI, marriage status, family history of myocardial infarction, smoking, educational status, systolic blood pressure and prevalent diabetes.
- *Model 2* AUC for algorithm including model 1 + paternal status (yes/no children).

AUCs were analysed for 5, 10, 15 and 20 years of follow-up. AUC was analysed by using ‘score.list’ in R package ‘riskRegression.

In the prospective analyses p-values were not included, instead 95% confidence intervals (CI) for measures of association were reported to display the measure of precision^25^. Statistical tests were performed using SPSS v.25 and SAS 9.4 (SAS University Edition).

## Results

From the 2572 men available for analysis 331 were *childless*, 1799 were *fathers*, and 442 were excluded from the analysis due to missing information. Mean (SD) follow up time was 18.9 (5.41) years. CVD was the primary cause in 35.2% of all deaths (327/930) until the end of follow up. The confirmatory cohort included 7008 fathers, 1180 childless men and 1360 were excluded due to missing information. Baseline characteristics for the exploratory and confirmatory cohort are presented in Table 1 and in Supplementary document.

**Table 1:** Baseline characteristics of the participants in the exploratory and confirmatory cohorts.

		Exploratory cohort		Confirmatory cohort	
		Childless men	Fathers	Childless men	Fathers
Age: mean (SD)		57.4 (6.1)	57.4 (5.9)	59.9 (7.4)	58.7 (7.8)
Educational level (N/%)	Did not complete elementary school	1/0.3%	13/0.8%	13/1.1%	49/0.8%
	Elementary school	157/50.3%	722/45.4%	567/48.2%	2807/43.2%
	Ground school (9–10 years of education)	66/21.2%	325/20.5%	204/17.3%	1289/19.9%
	High school	32/10.3%	193/12.1%	135/11.5%	795/12.2%
	At least one year after high school	24/ 7.7%	147/9.3%	106/9%	623/9.6%
	University degree	32/10.3%	189/11.9%	152/12.9%	929/14.3%
Smoking habits (N/%)	Yes, I smoke regularly	71/ 22.8%	335/21.1%	304/ 25.8	1413/21.7%
	Yes, I smoke occasionally	14/4.5%	83/5.2%	59/5%	307/4.7%
	No, I have stopped smoking	121/38.8%	688/43.3%	439/37.2%	2929/45%
	No, I have never smoked	106/34%	484/30.4%	377/32%	1861/28.6%
BMI: mean (SD)		26.1 (4.13)	26,1 (3.2)	26.1 (3.9)	26.4 (3.4)
Prevalent diabetes	No	281/90.1%	1546/94.9%	1090/92.4%	6646/94.8%
	Yes	31/ 9.9%	83/ 5.1%	90/7.6	364/5.2%
Systolic blood pressure: mean (SD)		144 (19)	141 (17)	142 (20)	140 (19)

### Distribution of GRS according to fatherhood status

**Figure 1:**
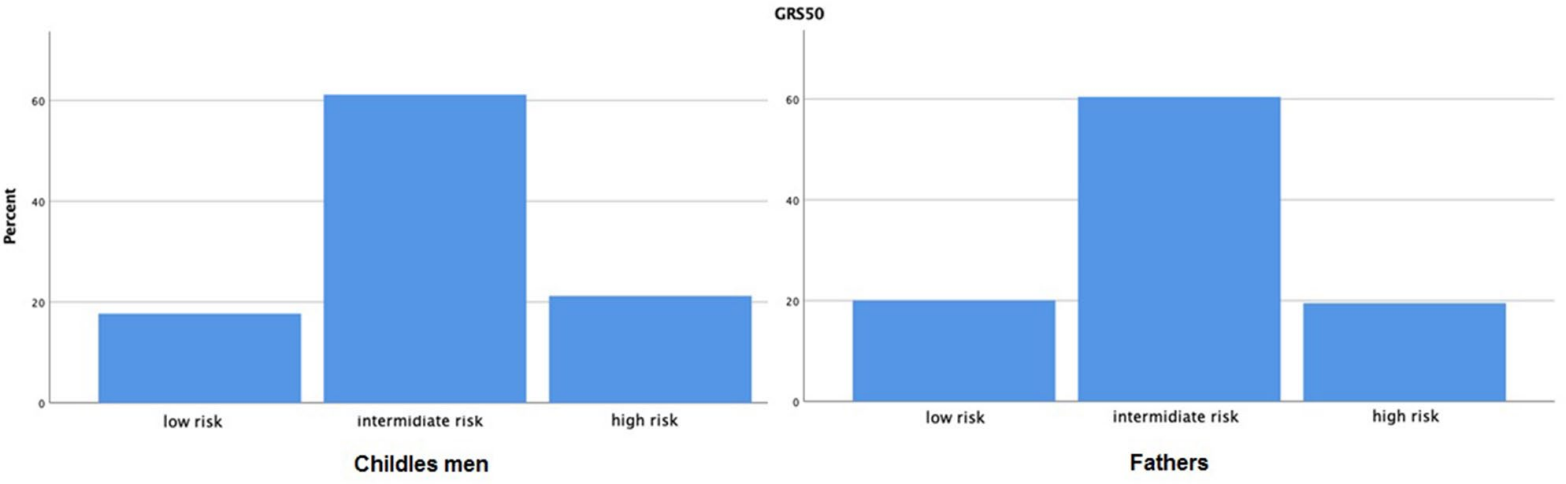
Bar plots representing the distribution of genetic risk groups for cardiovascular disease (GRS 50) between fathers and childless men.

There were no differences in the distribution of GRS 50 groups between *childless men* and *fathers.* (Table 2, Fig. 1). When using GRS50 as a continuous variable, no association with paternal status was seen (p = 0.73).

**Table 2:** Odds ratios (OR) for intermediate and high genetic risk scores (GRS 50) for CVD death in *childless* men as compared to *fathers* (reference). MDC—CVC cohort.

	OR (95% CI)*	p-value*	OR (95% CI)**	p-value**
Intermediate GRS	0.83 (0.58–1.18)	0.29	0.75 (0.48–1.15)	0.18
High GRS	0.82 (0.54–1.26)	0.36	0.78 (0.46–1.33)	0.37

*Crude analysis.

**Models adjusted for: age, smoking, alcohol, BMI, hypertension, family history of CI, cholesterol, triglycerides, marriage status.

#### Interaction between GRS and fatherhood status in relation to cardiovascular risk

In the combined strata the p-values for interaction between + /− fatherhood and low/intermediate/high GRS were non-significant—between 0.29 and 0.49 for CVD mortality and 0.38–0.76 for CAD in models that were adjusted for established cardiovascular risk factors.

### CVD mortality

**Figure 2:**
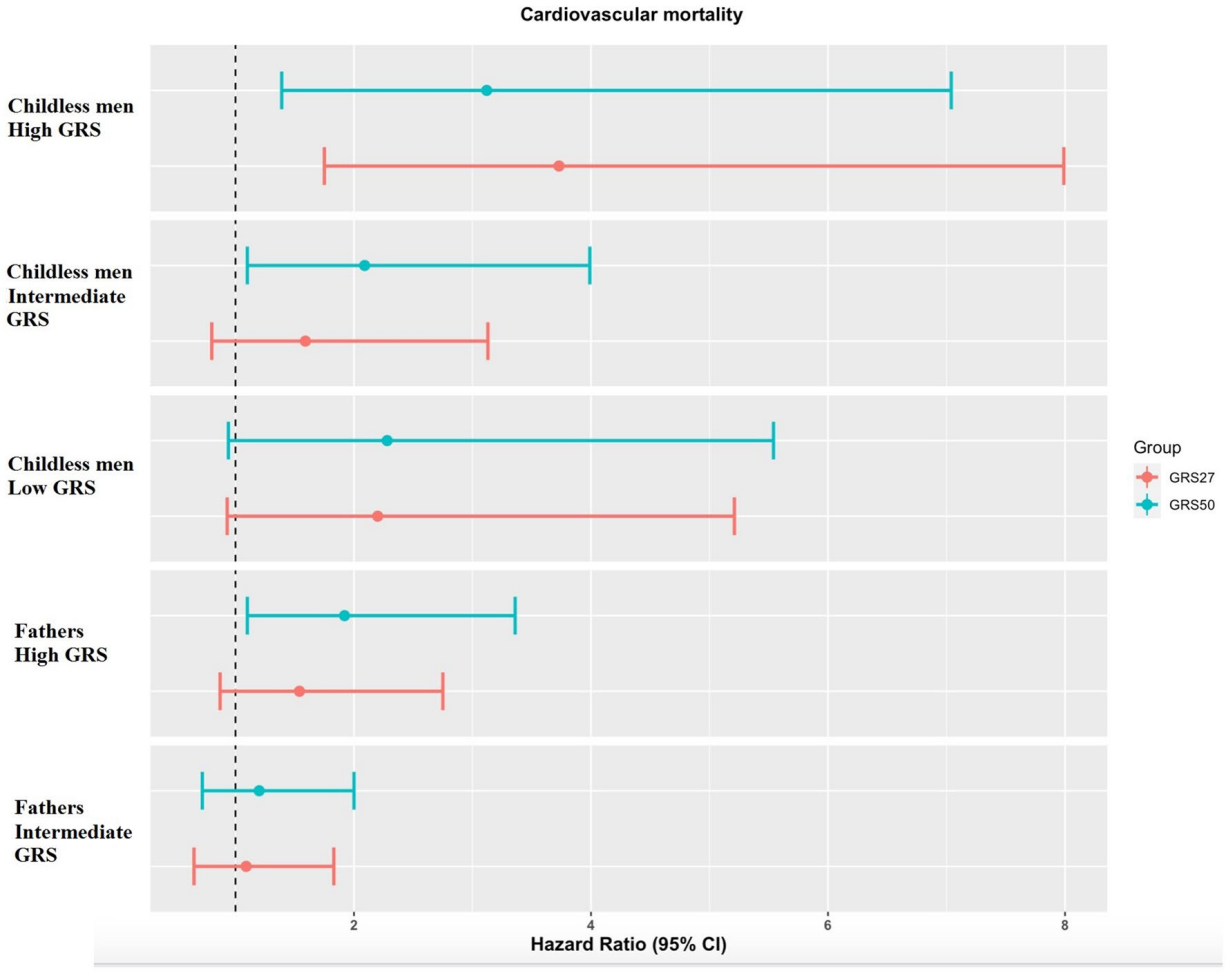
Competing risk regression for cardiovascular mortality among study groups using age as underlying timescale. Death unrelated to CVD is regarded as competing risk. The model is adjusted for BMI, smoking, alcohol consumption, marital status, family history of CVD, educational level, diabetes, cholesterol, triglycerides. Data source—MDC-CVC cohort.

Both in *fathers* and in *childless* men, GRS 50 was a predictor of CVD mortality (Table 3, Fig. 2). In the low GRS score group, the *childless* men did not differ statistically from the reference group—HR 2.28 (95% CI 0.94–5.54). In the intermediate GRS group, the *fathers* did not differ statistically from the reference [HR 1.20 (95% CI 0.72–2.00)] whereas, for GRS50, *childless* men presented with more than two times higher risk for CVD related death [HR 2.09 (95% CI 1.10–3.99)].

**Table 3:** Risk of cardiovascular (CVD) mortality and coronary artery disease (CAD) among study groups with GRS 50 with corresponding hazard ratios (HR) and 95% confidence intervals (95% CI). Figures in bold indicate statistical significance in MDC—CVC (exploratory) and MDC (confirmatory) cohorts.

Risk groups		CVD mortality		CAD	
Fatherhood	GRS	Exploratory cohort*	Confirmatory cohort**	Exploratory cohort*	Confirmatory cohort**
Fathers	Low	Reference	Reference	Reference	Reference
	Intermediate	1.20 (0.72–2.00)	**1.26 (1.05–1.50)**	**2.18 (1.47–3.23)**	**1.39 (1.19–1.62)**
	High	**1.92 (1.10–3.36)**	**1.31 (1.05–1.62)**	**2.53 (1.63–3.91)**	**2.07 (1.74–2.45)**
Childless men	Low	2.28 (0.94–5.54)	**1.57 (1.08–2.29)**	1.60 (0.69–3.73)	1.32 (0.93–1.90)
	Intermediate	**2.09 (1.10–3.99)**	**1.92 (1.50–2.46)**	**1.93 (1.14–3.27)**	**1.76 (1.40–2.20)**
	High	**3.12 (1.39–7.04)**	**2.62 (1.88–3.65)**	1.96 (0.96–3.98)	**3.02 (2.29–3.97)**

Models are adjusted for:

*Exploratory analysis: age, smoking, alcohol intake, BMI, hypertension, family history of CVD, cholesterol, triglycerides, and marital status.

**Confirmatory analysis: age smoking educational status, BMI, marital status, family history of CVD, prevalent diabetes.

High genetic risk was a predictor for CVD mortality especially pronounced among *childless* men, HR: 3.12 (95% CI 1.39–7.04) but also among *fathers* HR 1.92 (95% CI 1.10–3.36), Table 3.

The confirmatory analysis showed similar trend with highest risk estimates for CVD mortality among childless men with high GRS, HR: 2.51 (95% CI 1.86–3.36) for GRS 50 and HR 2.04 (95% CI 1.52–2.74) for GR 27, respectively, Table 3.

### CAD

Intermediate genetic risk was a predictor for CAD in both *fathers* and *childless* men, HR: 2.18 (95% CI 1.47–3.23) for *fathers,* and HR: 1.93 (95% CI 1.14–3.27) or *childless* men, Table 2. High GRS50 was a predictor of incident CAD only among the *fathers,* HR 2.53 (95% CI 1.63–3.91). These statistically significant results were also seen in the analysis based on the confirmatory cohort (Table 3).

The risk estimates for CAD and CVD mortality using GRS 27 were similar and are provided in the Supplementary document.

### Predictive effect of childlessness on CVD mortality

**Figure 3:**
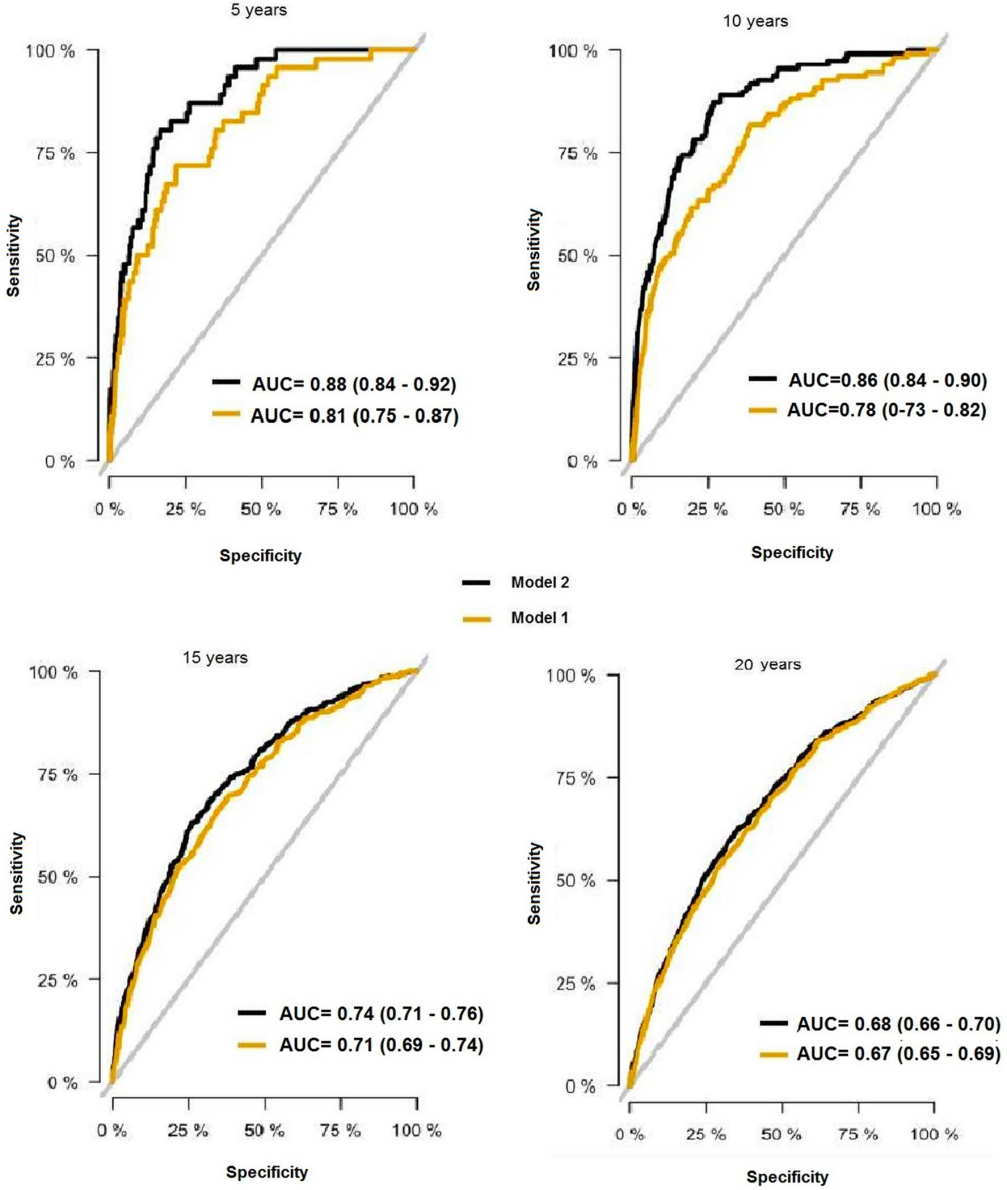
ROC curves for prediction of CVD mortality after 5-, 10-, 15- and 20-years follow-up. AUCs are given with 95% confidence intervals in brackets. *Model 1:* GRS 50 (continuous variable): adjusted for age, BMI, marriage status, family history of myocardial infarction, smoking, educational status, systolic blood pressure and prevalent diabetes. *Model 2:* GRS + paternal status (yes/no children): adjustments as in *Model 1.*

Algorithms including paternal information and GRS were more predictive for CVD mortality especially pronounced for 5 and 10 years, AUC 0.88 (95% CI 0.84–0.92) and 0.86 (95% CI 0.84–0.90), respectively, when compared to algorithms including only GRS without paternal status information, AUC 0.81 (95% CI 0.75–0.87) and 0.78 (95% CI 0.73–0.82), respectively (Fig. 3).

## Discussion

We found that the previously established increased CVD and mortality risk among childless men^4,26–28^ cannot be explained by those two types of conditions sharing predisposing genetic risk factors combined in GRS. Furthermore—in relation the cardiovascular outcomes—there was no statistically significant interaction between level of inherited genetic risk and state of fatherhood, implying that they contribute independently to the disease risk. On the other hand, as in the general population, being childless added to the risk of these unfavorable health outcomes in men being at increased risk due to intermediate or high GRS. This risk was most pronounced in childless men with high genetic risk scores having up to more than three times increased risk of CVD mortality, as compared to fathers with low GRS. Furthermore, we showed that adding information on paternal status to the GRS-based risk algorithm increases the predictive power for CVD mortality during 5- and 10-years follow-up.

Previously, by using data from a prospective cohort of 22,000 men with long follow-up from the same urban population, we were able to show that childlessness can be regarded as an independent risk marker for CVD along with other well-known risk factors^2^, an association previously reported by other authors^4,26–28^. In the current study we used a similar cohort from the same region of southern Sweden which provided genetic data and was specifically designed to study CVD risk. We were able to show the same effect of paternal status on CVD mortality estimates as previously published^2^. To the best of our knowledge this is the first study which evaluates the impact of established genetic risk scores for CVD on the association between parental status and the risk of CAD and CVD mortality.

Family size can be directly linked to the male fertility status^29^ and therefore male infertility is most likely overrepresented among childless men. Similarly, increased mortality and morbidity risk have been associated with impaired semen quality^2^ suggesting biological factors related to fertility to also play an important role for the risk of adverse health events in those men—association already established for women^30^.

The hypothesis of shared genetic traits for CVD and male infertility is based on the model proposed by Skakkebaek et al*.*^18^. It suggests a common mechanism, involving a combination of prenatal life exposures and adverse genetic factors to affect the future health of male fetuses making them more prone to develop subfertility as well as various diseases in adult life and to have shorter life span^16–19^. The mechanism suggests a primary testicular dysfunction including low testosterone—hypogonadism—as a possible mediator for the aforementioned risks^31,32^. Since up to 15% of the genome is directly involved in the physiology of reproduction^16^, disruption of non-reproductive, including metabolic, pathways likely impacts reproductive function and vice versa. However, the lack of interaction between parental status and inherited genetic risk for CVD reported by us suggests independent mechanisms when using childlessness as proxy for infertility.

Our study has several strengths but also some limitations. Comprehensive information from Swedish national registries allows for precise information on date and cause of death, emigration, disease diagnosis and represents men from all socioeconomic backgrounds. The meticulous data collection at baseline provides an opportunity to adjust for a large number of well-known risk factors for CVD. Furthermore, the genetic scores used in the analysis were previously verified as a risk factor using data from more than 55,000 individuals^15^, thus making it a reliable factor in risk estimation.

The robustness of our findings is underlined by confirmation of the findings based on MDC-CVC sub-cohort in the analysis of data from the remaining MDC subjects. In the latter analysis some additional GRS 27 subgroups showed statistically significantly increased CAD HRs, probably due to larger sample size.

One major limitation is that some of the six subgroups were quite small, implying a risk for type 2 error. Another limitation is the fact that GRS construction in the current study, compared to other recent reports, is based on a relatively low number of SNPs. Using larger number of polymorphisms^33^, based on large datasets such as UK biobank, have provided more than one GRS for CAD. The mounting number of GWAS studies in recent years has led to utilisation of hundreds of different SNPs, leading to construction of new GRSs able to outperform the older scores^34^. Therefore, it is plausible that the GRS used in the present study could be outperformed if a broader set of polymorphisms was utilised.

Furthermore, we could neither account for the participants´ paternal intention, nor the partner’s fertility status. Therefore, the number of children cannot be regarded as an optimal proxy for a man’s reproductive potential. Men who have adopted children or have fathered offspring with the help of assisted reproduction techniques, might have been included in the control group leading to definition- and selection bias. However, adoption rates in Sweden for the study period were relatively low, and assisted reproduction technologies efficient in cases of impaired male fertility were not available before 1992^34–37^. Therefore, this is unlikely to be a major source of bias.

Using childlessness at time of inclusion in MDC as marker of infertility could be questioned. In Sweden, current mean (SD) paternal age at the time of childbirth is 32.8 (6.2) years, this age being even lower at the time of baseline investigation for this study more than 25 years ago. The mean age at baseline was 57 years, which means that the vast majority of those who ever became fathers had one or more children at the time of inclusion in the study. Only one in four childless men in Sweden has been reported to be voluntary childless in the 1970s^35^, therefore the risk of our cohort to reflect voluntary childlessness is low. Furthermore, since infertility is a couple problem (disease) and reliable biomarkers for evaluation of fertility are lacking, alternative methods of assessment of male reproductive capacity, e.g. semen analysis and/or endocrine evaluation also possess a number of shortcomings.

The end points used in this study are influenced by biological as well as social factors. Lonely fathers have been found to be at higher risk of CVD and mortality than cohabiting fathers^28^, therefore the lack of information on how long the children lived with their fathers and in what family situation is another limitation. Marital strain is also unaccounted for while at the same time this is a factor that might influence the number of children, and also independently relate to a higher risk of CVD, and premature mortality^38^. Nevertheless, these types of bias related to family dynamics, fertility, and other unaccountable factors would rather have led to misclassification between the study groups, and thereby bias toward the null hypothesis.

Furthermore, in a prospective study, detecting a risk factor/marker at the baseline examination might have given the opportunity to some of the men to adjust their lifestyle or receive treatment, thereby possibly influencing their risk of developing MACE and to die from CVD. It is unclear if this has had any significant effect on our results, but previous research has pointed to the fact that adherence to a healthy lifestyle might reduce the risk in men with high and intermediate GRS^15^.

The results of this observational study support two noteworthy conclusions. *Firstly*, our analysis suggests that inherited DNA variation and childlessness contribute independently of each other to the susceptibility for CVD and related mortality. *Secondly*, being childless is associated with a similar risk increase for cardiovascular events across every stratum of genetic risk. The biological explanation for the observed associations remains to be determined. Nevertheless, on the verge of the era of personalised medicine, information on inherited risk for CVD can be known as early as at birth by genetic testing. Reproductive status can also be estimated in young adulthood—often many years before the development of CVD. Combining information on fertility status and GRS might identify a small group of young men who—compared to the rest of population and even after accounting for the other well-known risk factors—have significantly increased risk of fatal CVD. Various preventive measures might be considered in this group aiming to improve the lifespan and quality of life.

## Conclusion

Men have shorter lifespan than women at present in Western countries and do, generally, to a less degree than women, consume health care for disease preventing measures. The subgroup of men with fertility problems represents a significant proportion of the general male population. Improving the risk assessment algorithms for CVD by including more risk factors/markers such as reproductive history (fertility status) is an important public goal, as already proposed for women. This analysis showed that combining information on parental status and inherited risk for CVD may improve the predictive power of risk algorithms in middle-aged men. Whereas further studies are needed to establish which particular groups of childless or sub-fertile men possess the highest health risks and would benefit from early counselling and preventive measures, childless men and those with severe infertility problem may be an important target group for prevention of CVD.

## Supplementary Information

Supplementary Information.
