# Supplementary material for: Impact of genetic risk score on the association between male childlessness and cardiovascular disease and mortality
Source: Sci Rep. 2021 Sep 17;11:18526. doi: 10.1038/s41598-021-97733-2 (PMC8448891; doi:10.1038/s41598-021-97733-2)
Supplement: Supplementary file 1 — Supplementary Information. [file 41598_2021_97733_MOESM1_ESM.docx]

**Impact of Genetic Risk Score on the Association between Male Childlessness and Cardiovascular Disease and Mortality**

**Angel Elenkov ^1,2^, Olle Melander ^3,4^, Peter M Nilsson ^3,4^, He Zhang^2^, Aleksander Giwercman ^1,2^**

**Supplementary document**

**Modelling of genetic risk score 27 (GRS27).**

The 27-SNP GRS (GRS27) is described by Mega et al. The GRS of each individual was calculated as follows: the previously reported risk estimate for the modelled allele of each SNP was natural log-transformed and multiplied by two for homozygotes and with one for heterozygotes. The products were then summed. The mean (2.49 for GRS27) and standard deviation (0.34 for GRS27) of the study population were used to standardize each GRS to have a mean of 0 and one SD as unit of variance. Genetic risk was analyzed per standard deviation increment of the standardized GRS as well as by comparing those with *high* GRS (Quintile 5), with those with *intermediate* risk score (Quintiles 2 to 4), and those with *low* GRS (Quintile 1).

More detailed information on reported risk estimates for the individual SNPs is freely available at: [*https://www.nejm.org/doi/suppl/10.1056/NEJMoa1605086/suppl_file/nejmoa1605086_appendix.pdf*](https://www.nejm.org/doi/suppl/10.1056/NEJMoa1605086/suppl_file/nejmoa1605086_appendix.pdf)

**Results**

***Distribution of GRS 27 according to fatherhood status.***

There were no differences in the distribution of GRS 27 groups between *childless men* and *fathers.*  (**Table 2**). When using GRS as a continuous variable, no difference was established either (p=0.97).

***CVD mortality***

Both in *fathers* and in *childless* men, GRS 27 was a predictor of CVD mortality (**Table 3, Figure 2**). In the low GRS score group, the *childless* men did not differ statistically from the reference group HR: 1.60 (95%CI: 0.69 – 3.73). In the intermediate GRS group, the *fathers* did not differ statistically from the reference HR: 1.09 (95%CI: 0.65 – 1.83), as did not the *childless* men (HR: 1.59 (95%CI: 0.80 - 3.13)).

High genetic risk was a predictor for CVD mortality especially pronounced among *childless* men HR: 3.73 (95%CI: 1.75 - 7.99)) for GRS27), but not for fathers (HR: 1.54 (95%CI: 0.87 - 2.75, **Table 3**).

The confirmatory analysis showed similar trend with highest risk estimates for CVD mortality among childless men with high GRS (HR:2.51;(95%CI:1.86 – 3.36 for GRS 50 and HR:2.04;95%CI:1.52 – 2.74 for GR 27, respectively. Table 3).

***CAD***

High GRS27 was a predictor among *childless* men (HR: 2.11(95%CI: 1.06 – 4.21)). These statistically significant results were also seen in the analysis based on the confirmatory cohort, which additionally showed increased HR for fathers with high GRS 27 and childless men with intermediate GRS 27 (Table 3).

**Table 1.** Baseline characteristics among study groups for GRS27

| **Risk groups** |  | **AGE:**  **Years**  **Mean (SD)** | **Alcohol:**  **g/day**  **Mean (SD)** | **BMI**  **kg/m^2^**  **Mean (SD)** | **Glucose**  **nmol/L**  **Mean (SD)** | **HDL**  **nmol/L**  **Mean (SD)** | **LDL**  **nmol/L**  **Mean (SD)** |
| --- | --- | --- | --- | --- | --- | --- | --- |
| **Fatherhood**  **status** | **GRS** | **GRS 27** | **GRS 27** | **GRS 27** | **GRS 27** | **GRS 27** | **GRS 27** |
| Fathers | Low | 56.8 (6.1) | 14.7 (13.09) | 26.1 (3.3) | 5.29 (1.4) | 1.24 (0.31 | 4.04 (0.86 |
|  | Intermediate | 57.1 (5.7) | 116.0 (15.25) | 26.1 (3.2) | 5.3 (1.5) | 1.2 (0.3) | 4.1 (0.9) |
|  | High | 57.0 (6.1) | 15.8 (15.1) | 26.1 (3.3) | 5.29 (1.1) | 1.2 (0.3) | 4.2 (0.9) |
| Childless  men | Low | 57.5 (6.5) | 16.4 (19.3) | 26.2 (4.1) | 5.55 (1.3) | 1.20 (0.4) | 4.1 (1.0) |
|  | Intermediate | 57.5 (6.1) | 13.9 (18.1) | 26.1 (4.23) | 5.57 (1.9) | 1.18 (.0.28) | 4.1 (0.9) |
|  | High | 56.3 (5.9) | 14.7 (21.4) | 25.7 (3.8) | 5.31 (1.4) | 1.32 (0.4) | 4.1 (0.9) |

**Table 2.** Odds ratios (OR) for intermediate and high genetic risk scores (GRS 27) for CVD death in *childless* men as compared to *fathers* (reference). MDC – CVC cohort.

|  | OR (95%CI)* | p-value* | OR (95%CI)** | p-value** |
| --- | --- | --- | --- | --- |
| Intermediate GRS | 1.03 (0.72 – 1.45) | 0.87 | 0.84 (0.54 – 1.29) | 0.41 |
| High GRS | 1.03 (0.69 – 1.64) | 0.75 | 0.87 (0.51 – 1.52) | 0.64 |

*Crude analysis

**Models adjusted for: age, smoking, alcohol, BMI, hypertension, family history of CI, cholesterol, triglycerides, marriage status.

**Table 3.** Baseline characteristics among study groups for GRS 50 in the exploratory cohort.

| **Risk groups** | | **AGE:**  **Years**  **Mean (SD)** | **Alcohol:**  **g/day**  **Mean (SD)** | **BMI**  **kg/m^2^**  **Mean (SD)** | **Glucose**  **nmol/L**  **Mean (SD)** | **HDL**  **nmol/L**  **Mean (SD)** | **LDL**  **nmol/L**  **Mean (SD)** |
| --- | --- | --- | --- | --- | --- | --- | --- |
| **Fatherhood**  **status** | **GRS** |  |  |  |  |  |  |
| Fathers | Low | 57.19 (6.09) | 14.7 (13.8) | 26.0(3.3) | 5.3 (1.2) | 1.24(0.3) | 4.07(0.89) |
|  | Intermediate | 56.9 (5.7) | 16.1 (14.9) | 26.0 (3.1) | 5.3 (1.5) | 1.21 (0.3) | 4.1 (0.9) |
|  | High | 57.3 (6.2) | 15.6 (15.6) | 26.3 (3.5) | 5.3 (1.1) | 1.19 (0.3) | 4.1 (0.9) |
| Childless  men | Low | 57.8 (6.2) | 13.2 (13.9) | 26.2 (3.2) | 5.43 (1.3) | 1.25 (0.4) | 4.09 (1.0) |
|  | Intermediate | 57.6 (6.1) | 14.34 (19.2) | 26.1 (4.4) | 5.59 (1.9) | 1.18 (0.3) | 4.1(0.9) |
|  | High | 55.9 (6.0) | 16.2 (21..9) | 25.8 (4.0) | 5.34 (1.3) | 1.30 (0.4) | 4.23 (0.9) |

**Table 3.** Risk of cardiovascular (CVD) mortality and coronary artery disease (CAD) among study groups with GRS 27 with corresponding hazard ratios (HR) and 95% confidence intervals (95%CI). Figures in bold indicate statistical significance in MDC – CVC (exploratory) and MDC (confirmatory) cohorts.

| **Risk groups** | | **CVD mortality** | | **CAD** | |
| --- | --- | --- | --- | --- | --- |
| Fatherhood | GRS | **Exploratory cohort** | Confirmatory cohort | **Exploratory cohort** | Confirmatory cohort |
| Fathers | Low | *Reference* | *Reference* | *Reference* | *Reference* |
|  | Intermediate | 1.09 (0.65 - 1.83) | **1.12 (0.95 – 1.34)** | **1.87 (1.26 – 2.81)** | **1.19 (1.03 – 1.38)** |
|  | High | 1.54 (0.87 - 2.75) | **1.25 (1.02 – 1.55)** | 2.23 (1.43 – 3.48) | **1.76 (1.49 – 2.07)** |
| Childless men | Low | 2.20 (0.93 - 5.21) | **1.48 (1.02 – 2.15)** | 1.33 (0.56 – 3.17) | 1.34 (0.96 – 1.87) |
|  | Intermediate | 1.59 (0.80 - 3.13) | **1.85 (1.45 – 2.37)** | 1.66 (0.98 – 2.83) | **1.51 (1.21 – 1.89)** |
|  | High | **3.73 (1.75 - 7.99)** | **2.08 (1.50 – 2.89)** | **2.11 (1.06 – 4.21)** | **2.43 (1.85 – 3.19)** |

Models are adjusted for:

Exploratory analysis: age, smoking, alcohol intake, BMI, hypertension, family history of CVD, cholesterol, triglycerides, and marital status.

Confirmatory analysis: age smoking educational status, BMI, marital status, family history of CVD, prevalent diabetes
